# Characterization of the Domoic Acid Uptake Mechanism of the Mussel (*Mytilus galloprovincialis*) Digestive Gland

**DOI:** 10.3390/toxins13070458

**Published:** 2021-06-30

**Authors:** Juan Blanco, Carmen Mariño, Helena Martín, Gonzalo Álvarez, Araceli E. Rossignoli

**Affiliations:** 1 Centro de Investigacións Mariñas (CIMA), Xunta de Galicia, Pedras de Coron s/n, 36620 Vilanova de Arousa, Spain; maria.carmen.marino.cadarso@xunta.gal (C.M.); helena.martin.sanchez@xunta.gal (H.M.); araceli.escudeiro.rossignoli@xunta.gal (A.E.R.); 2 Departamento de Acuicultura, Universidad Católica del Norte, Larrondo 1281, Coquimbo, Chile; gmalvarez@ucn.cl; 3 Centro de Investigación y Desarrollo Tecnológico en Algas y Otros Recursos Biológicos (CIDTA), Facultad de Ciencias del Mar, Universidad Católica del Norte, Larrondo 1281, Coquimbo, Chile

**Keywords:** membrane transporter, uptake velocity, sodium-independent, chloride-dependent, accumulation, maximum levels, cyanide, pH, *Pseudo-nitzschia* cell quota

## Abstract

Cultures of the mussel *Mytilus galloprovincialis* are frequently affected by accumulation of the amnesic shellfish poisoning toxin domoic acid (DA). This species is characterized by a fast uptake and release of the toxin. In this work, the main characteristics of the uptake mechanism have been studied by incubation of digestive gland thin slices in media with different composition and DA concentration. DA uptake seems to follow Michaelis–Menten kinetics, with a very high estimated K_M_ (1722 µg DA mL^−1^) and a V_max_ of 71.9 µg DA g^−1^ h^−1^, which is similar to those found for other amino acids in invertebrates. Replacement of NaCl from the incubation media by Cl-choline (Na^+^-free medium) did not significantly reduce the uptake, but replacement by sorbitol (Na^+^-free and Cl^−^-depleted medium) did. A new experiment replacing all chlorides with their equivalent gluconates (Na^+^- and Cl^−^-free medium) showed an important reduction in the uptake that should be attributed to the absence of chloride, pointing to a Na^+^-independent, Cl^−^ (or anion-) dependent transporter. In media with Na^+^ and Cl^−^, neither decreasing the pH nor adding cyanide (a metabolic inhibitor) had significant effect on DA uptake, suggesting that the transport mechanism is not H^+^- or ATP-dependent. In a chloride depleted medium, lowering pH or adding CN increased the uptake, suggesting that other anions could, at least partially, substitute chloride.

## 1. Introduction

Domoic acid is a tricarboxylic amino acid first isolated from the red alga *Chondria armata* [1]. In 1987, it was identified as the main substance responsible [2] for a number of cases of intoxication caused by consumption of mussels grown in Prince Edward Island, Canada [3,4]. The syndrome associated with the intoxication was characterized mainly by the loss of recent memory, which led to it being named Amnesic Shellfish Poisoning (ASP) [5]. Two years after the first intoxication, the source of domoic acid in the plankton was identified as the pennate diatom *Nitzschia pungens* (currently *Pseudo-nitzschia multiseries*) [6]. Since then, populations of toxic *Pseudo-nitzschia* and the presence of domoic acid in bivalves have been reported worldwide [7,8,9,10,11,12,13,14,15,16,17].

Domoic acid accumulation in bivalves varies substantially between species. It is known that this variability is in part due to differences in depuration velocity. Some bivalves depurate most of the toxin very quickly, as is the case for the mussels *M. galloprovincialis* [18], *M. edulis* [19,20], *Mesodesma donacium* [21], and the scallop *Argopecten purpuratus* [22], while other species can retain it for very long periods of time, such as the razor clam *Siliqua patula* [23,24,25] or the king scallop *Pecten maximus* [26,27,28]. Mafra et al. [29,30] showed that the reduction in the amount of toxic cells ingested by pre-ingestive selection of the phytoplankton species can partially explain the observed differences between bivalve species, but the role of toxin absorption mechanisms in accumulation (acting after toxin ingestion) has not been sufficiently studied.

The presence of domoic acid in marine organisms, and especially in bivalves, produces important losses to fisheries and aquaculture because harvesting and marketing is banned when the DA concentrations exceed the established maximum allowable level, which in European Union and most countries is currently 20 mg kg^−1^ of soft tissues [31]. Furthermore, the high risk of persistent DA contamination discourages attempts to culture certain species of high commercial interest, such as *Pecten maximus*, because of the high risk of not being able to market it for months or even years if they are exposed to a toxic *Pseudo-nitzschia* bloom [26,32].

Developing methods to reduce the DA absorption, by chemical or biological treatments, genetic selection, or others, would be very useful for minimizing the consequences of *Pseudo-nitzschia* blooms. To effectively develop those methods, it is necessary to know the mechanism by which the toxin is taken up by the digestive gland of the bivalves (the main organ by which DA is incorporated from particles [20]), which is not currently known. Furthermore, very little information about DA absorption by bivalves exists.

In the digestive gland of the mussel *M. edulis*, the absorption has been suggested to take place by a cellular membrane transporter characterized by a marginal dependence on ATP and by the competitive inhibition of domoic acid intake by some structurally related amino acids, like kainic and glutamic acids, or proline [33].

In this work, we attempted to characterize in detail the mechanism of absorption of DA in the digestive gland of the mussel *M. galloprovincialis*. In a series of experiments, the implications of a transporter, sodium, chloride, pH, and energy have been checked in order to confirm that a transporter is involved, to contribute to its identification, and to ascertain the uptake kinetics.

## 2. Results

### 2.1. Domoic Acid Uptake Velocity and Saturation of the Transport

**Figure 1:**
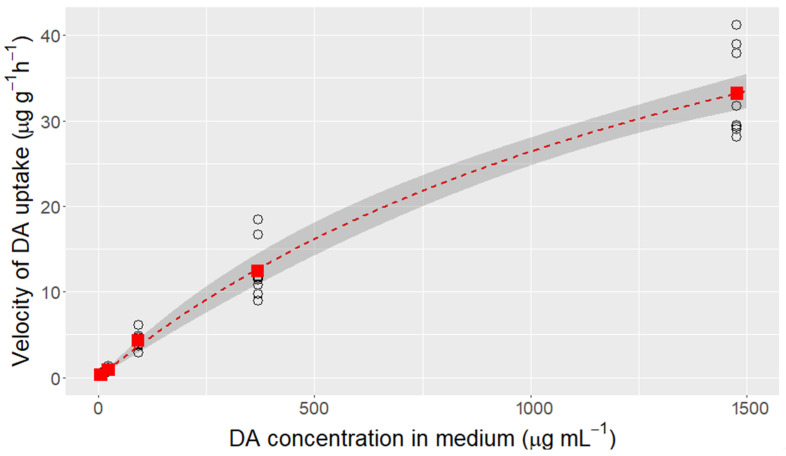
Domoic acid uptake rate by mussel digestive gland slices as a function of the DA concentration in the incubation medium. Circles are the actual values for each slice, solid squares are the means for each concentration and the dotted line is the fitted Michaelis–Menten kinetics. The shadowed area represents the 95% confidence interval for the fitted kinetics.

The uptake of domoic acid from seawater by the mussel’s digestive gland followed Michaelis–Menten kinetics (at least in its initial part) but the maximum concentration used in this study was still well below the saturation level. The estimated V_max_ and K_m_ were 71.9 µg DA g^−1^ h^−1^ and 1722 µg DA mL^−1^, respectively (Figure 1).

### 2.2. Effect of Environmental Sodium

**Figure 2:**
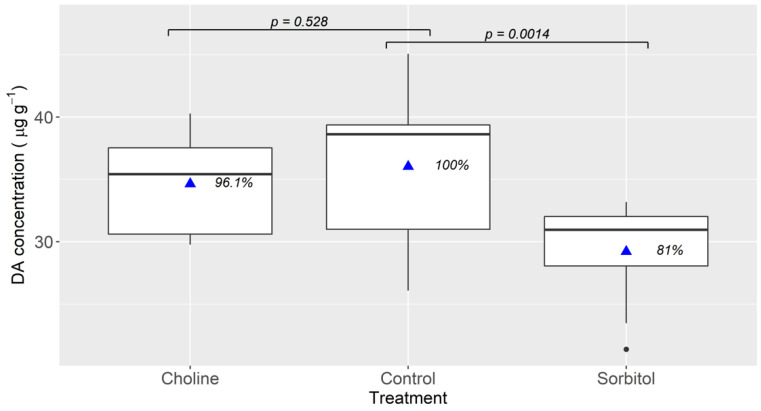
Accumulation of domoic acid in mussel digestive gland tissues after 3 hours of incubation in seawater (control) and in two other media in which NaCl was replaced by choline chloride and sorbitol. The upper and lower limits of the boxes are the quartiles, the middle horizontal line is the median, the extremes of the vertical lines are the upper and lower limits of the observations, the dots are the outliers (values that deviate from the median more than 1.5 times the interquartile range), the triangles are the mean and the numbers to the right of the triangles are the percentages of the control treatment. The horizontal segments with associated probability values indicate the significance of the difference between the treatments at the extremes of the segments (paired Student *t*-test).

The results obtained when the incubation media did not contain Na were different depending on the compound which was used to replace NaCl. When choline (added to the medium as Cl-choline) was used, no significant reduction in the uptake was observed, while when sorbitol was used it was significantly reduced (Figure 2).

### 2.3. Effects of Cyanide in a Chloride-Depleted Environment and pH

**Figure 3:**
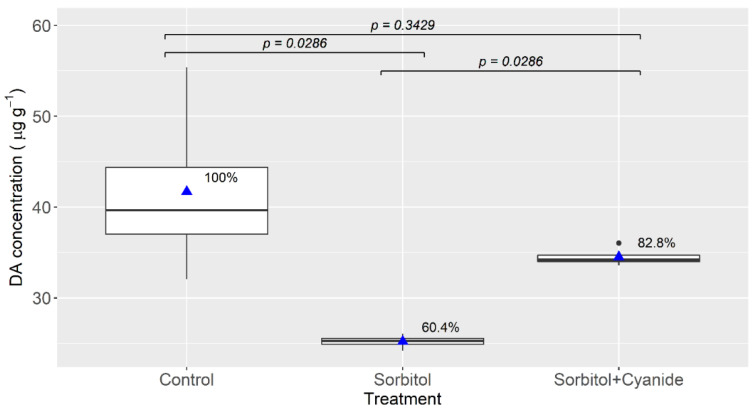
Accumulation of domoic acid in mussel digestive gland tissues after 3 hours of incubation in seawater (control), and in two other media with NaCl replaced by sorbitol, one of which was supplemented with sodium cyanide. The upper and lower limits of the boxes are the quartiles, the middle horizontal line is the median, the extremes of the vertical lines are the upper and lower limits of the observations, the dots are the outliers, the triangles are the mean, and the numbers to the right of the triangles are the percentages of the control treatment. The horizontal segments with associated probability values indicate the significance of the difference between the treatments at the extremes of the segments (Wilcoxon test).

When cyanide was added to the sorbitol medium (chloride-depleted), a significant increase in the DA uptake, compared to the sorbitol medium alone, took place. The final DA concentration in the slices incubated in this medium was nearly 83% of the one in the control (seawater without cyanide) (Figure 3).

**Figure 4:**
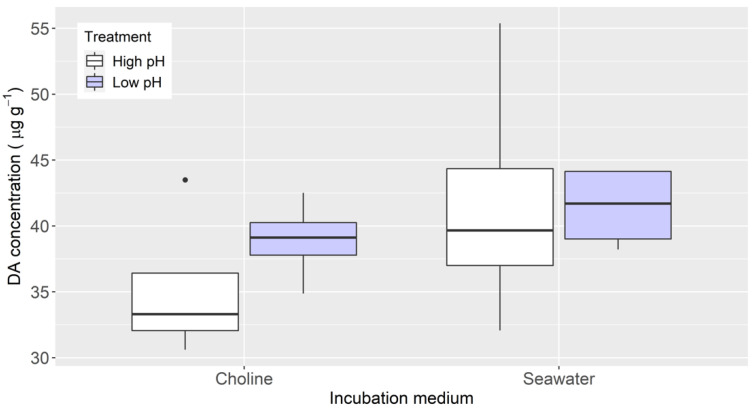
Accumulation of domoic acid in mussel digestive gland tissue after 3 hours of incubation in seawater with high and low pH, and a medium in which NaCl was replaced by choline chloride, also with high and low pH. The upper and lower limits of the boxes are the quartiles, the middle horizontal line is the median, the extremes of the vertical lines are the upper and lower limits of the observations, the dots are the outliers.

The pH changes did not significantly affect the DA uptake, neither in the “choline” nor in the “seawater” medium. In both cases, and especially in the “choline” medium, the slices absorbed more DA when the pH was lower (with H_3_PO_4_ added) (Figure 4).

### 2.4. Effect of Environmental Chloride and Cyanide

**Figure 5:**
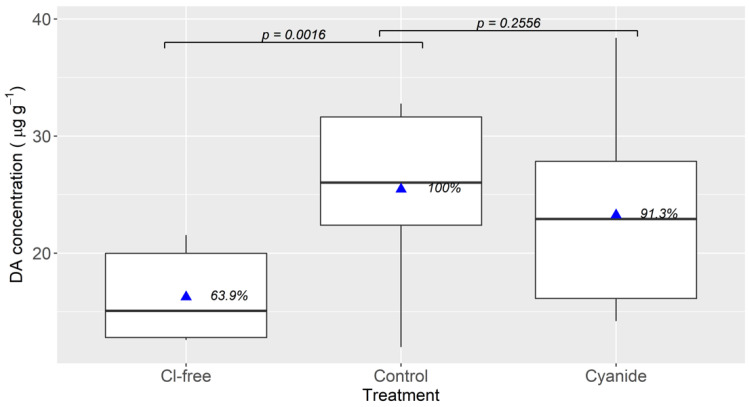
Accumulation of domoic acid in mussel digestive gland tissues after 3 hours of incubation in seawater (control), a medium in which all the chlorides were replaced by their corresponding gluconates (Cl-free), and in seawater to which the metabolic inhibitor sodium cyanide was added (cyanide). The upper and lower limits of the boxes are the quartiles, the middle horizontal line is the median, the extremes of the vertical lines are the upper and lower limits of the observations, the dots are the outliers, the triangles are the mean and the numbers to the right of the triangles are the percentages of the control treatment. The horizontal segments with associated probability values indicate the significance of the difference between the treatments at the extremes of the segments (paired Student *t*-test).

When all chlorides were omitted from the culture medium, DA uptake was significantly reduced, with the DA accumulated at the end of the experiment being 63.9% that of the control (Figure 5). This percentage was also lower than that obtained in the treatment with sorbitol in Section 4.3, in which some chlorides (but not NaCl) were present in the incubation medium.

The addition of cyanide to the control medium had a minor, and not statistically significant, effect on DA uptake by the digestive gland slices.

## 3. Discussion

The uptake of DA by the mussel digestive gland is not linear with the concentration in the environment. The data obtained effectively follows Michaelis–Menten kinetics, suggesting that the uptake is carried out by a transporter protein of the cell membrane. This mechanism has already been suggested by Madhyastha et al. [33] in light of the effect that some structurally-related amino acids had on the uptake velocity. The uptake parameters have not been precisely estimated because the DA concentrations used were not high enough to approach the asymptote of the Michaelis–Menten curve, but it is very likely that they constitute a good approximation.

The uptake velocity is of the same order of magnitude as those found for other amino acids in invertebrates, such as glycine and alanine [34] in mussel *M. edulis* larvae, L-valine in the annelid *Nereis virens* [35], glutamic acid in several *Nereis* species [36], or in the crab *Carcinus maenas* [37], and others in *Crassostrea gigas* [38].

In extreme, but possible, conditions during a *Pseudo-nitzschia* bloom, the transporter could be saturated because the DA concentrations attained in the digestive system could be much higher than the estimated K_m_. For example, assuming bloom of *P. australis* (the most toxic *Pseudo-nitzschia* species), in which the cells have a volume of 750 µm^3^ and a DA content of 25 pg (as found in some cultures [39]), and also assuming that 90% of the digestive system is occupied by disrupted cells, the DA concentration in the digestive system would be 17,333 µg mL^−1^., that is, more than 10 times higher than the estimated K_m_ for the DA uptake. Under these conditions, therefore, the uptake velocity would be maximal, and mussels could uptake DA at levels exceeding the regulatory limit in approximately 3 h (considering that the digestive gland of a mussel weighs approximately 1 g and represents 10% of the body weight, on average). Under more realistic conditions (using the highest estimates of *P. australis* biovolume (4084 µm^3^) [40], a DA cell quota of 4 pg cell^−1^, and a digestive system occupation of 50%), the uptake velocity would be approximately 16 µg DA g^−1^ h^−1^ and the mussels would take approximately 13 h to attain the regulatory limit concentration. These estimated times would be longer if some *Pseudo-nitzschia* cells pass through the digestive system intact, as happens with *Alexandrium* in the clam *Mercenaria* [41].

**Figure 6:**
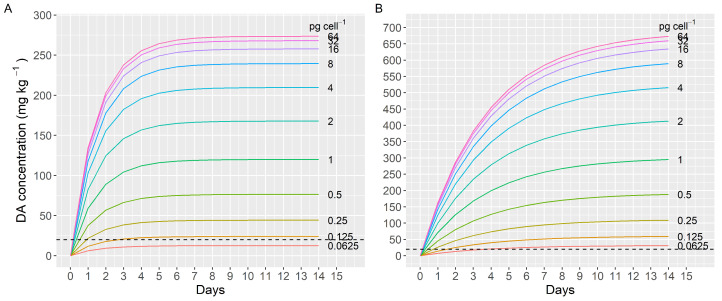
Simulated domoic acid accumulation in mussels, as a function of the *Pseudo-nitzschia* cell toxin content, assuming a precise cell volume, that all cells are completely filling the digestive system, and depuration rates of 0.68 (**A**), and 0.27 day^−1^ (**B**). The dashed line represents the regulatory level.

When longer periods of time are considered (14 days of continuous intoxication) and a depuration rate of 0.68 day^−1^ (recalculated from Blanco et al. [18]) is also taken into account, the simulation of the DA accumulation shows that the DA concentration in the mussels reaches a maximum, which increases very slowly with the toxin content of the *Pseudo-nitzchia* cells, of around 260 mg kg^−1^ (Figure 6). This value is consistent with the maximum value (248 mg kg^−1^) recorded in more than 25 years of monitoring in Galicia (data from Intecmar, publication in preparation). With toxin concentrations in the digestive system below those produced by a 100% occupation and DA per cell of approximately 0.1 pg, the toxin concentration in the mussel is not expected to reach the regulatory level (Figure 6A). It can be observed that an increase in cell toxin content above 16 pg has little effect on the maximum toxin accumulated by mussels with both depuration rates (Figure 6A,B). Notwithstanding this, those maximum values could be substantially affected by changes in the depuration rate.

In Prince Edward Island, Canada, where the first human intoxication by domoic acid was recorded, mussels of a closely related species, *M. edulis*, were found to contain 790 mg kg^−1^ of DA. Assuming that the characteristics of its DA transporter is similar to the one of *M. galloprovincialis*, and using the depuration rate for large starved mussels at 6 °C and salinity of 18 re-calculated from the data by Novaczek et al. [19], which is 0.46 day^−1^, the maximum attainable concentration would be around 400 mg kg^−1^. Those authors found a noticeable reduction in the depuration rate with temperature. From their data, we have estimated a rate of 0.27 day^−1^ for 3 °C, which is not unlikely for the surficial water of the area in November–December. In such a case, the maximum DA level that could be attained would be around 680 mg kg^−1^, which is also coherent, even than somewhat lower, with the existing data. In other species, as the king scallop *P. maximus* with much lower depuration rate, even if the transporter has the same characteristics, the accumulation would be expected to be substantially higher (Figure S1).

The transporter involved (or at least the main one) does not seem to be Na^+^-dependent, because the replacement of the salts containing Na by others containing K or choline chloride did not have any significant effect on DA uptake (Figure 2). The replacement of NaCl by sorbitol did reduce the uptake but, considering the results obtained when NaCl was replaced by choline chloride, the reduction should be attributed to the much smaller concentration of chloride in that medium (Cl^−^ was still present in the medium because some salts containing Cl^−^ were not replaced). Another experiment, carried out to confirm the effect of Cl^−^, in which Cl^−^-containing salts were replaced by their corresponding gluconates, showed that the uptake was significantly reduced, suggesting that the DA transport could be chloride-dependent, but not strictly dependent because the uptake in absence of Cl^−^ was not completely suppressed. The involvement of several transporters, while possible, seems unlikely because the uptake follows Michaelis–Menten kinetics and it would require that all the transporters involved had the same uptake parameters.

The small effect that the addition of cyanide had in the DA uptake when the tissues were incubated in seawater (sodium- and chloride-replete media) suggests that the transport is not directly ATP-dependent. The lack of effect of the pH under the same conditions also suggests that it is not H^+^-dependent. Nevertheless, these two treatments increased the uptake in chloride-depleted media. This opens the possibility that other anions could replace chloride; this aspect, as well as the dependence of the uptake on the intensity of the chloride gradient, deserves additional study. 

Most amino acid transporters are Na^+^-dependent [42,43,44,45,46,47]. Some Cl^−^-dependent amino acid transport systems have also been described, but most of them also require Na^+^ [42,48,49,50]. Na^+^-independent amino acid transporters exist [51] but are much less common, at least in mammals. Cl^−^-dependent glutamate transport has been described in the synaptic membrane [52]. It was hypothesized to be an exchange between glycine and glutamate and was induced by the energy shortage derived from ischemia, which would be consistent with the little effect that cyanide has on the domoic acid uptake. Sialin, the aspartate transporter described by Miyaji et al. [53], which is Na^+^-independent and Cl^−^-dependent, could be a possible candidate, but it was found to be overexpressed in mussel digestive gland tissue during domoic acid depuration [54], therefore, increasing its expression in the opposite direction to that expected if it was involved in the uptake. Another group of membrane proteins, the Organic Anion Transporters (OATs) which have some Cl^−^-dependent members, could be involved in the uptake of DA. The Organic Anion Transporter Proteins (OATPs), for example (that are involved in the hepatic function of mammals) can transport anions such as sulfobromophthalein (BSP) in a Na^+^-independent way but with important reduction in the rate when chloride is omitted from the incubation medium of rat hepatocytes [55,56]. The unequivocal identification of the transporter involved in DA uptake will require further study.

## 4. Materials and Methods

### 4.1. Biological Material and Sample Preparation

Mussels *M. galloprovincialis* from the Galician Rías were supplied by the Instituto Tecnolóxico para o Control do Medio Mariño (Intecmar, Vilagarcía de Arousa, Spain). 

For all experiments, the mussels were opened, dissected to isolate the digestive gland, and then 3 to 6 thin (less than 1 mm) slices of approximately 80–100 mg were obtained from the central part of the digestive gland by mean of transversal cuts. The small part of stomach epithelium which remained was dissected and discarded, keeping only the hepatopancreas tissues, which are responsible for absorption and internal digestion. Each slice was weighed and placed into a culture plate with 24 wells filled with 2 mL of filter-sterilized seawater and very gently shaken in an orbital shaker (the minimum speed which produced an observable movement of the slices). Once all the needed slices were weighed and washed, they were then rinsed and washed again with seawater and transferred to other 24-well plates containing the incubation media corresponding to the treatments in each experiment. In Section 4.2, the slices were attributed to the treatments randomly. In all other experiments, slices of the same mussels were assigned to each treatment. Additionally, one slice of each mussel was extracted with 50% MeOH to quantify the initial DA concentration. The slices were incubated for 3 h, in the dark, at room temperature (20–25 °C), while subjected to gentle agitation using an orbital shaker.

At the end of the incubation period, the slices were placed in Eppendorf tubes filled with isotonic ammonium formate and centrifuged at 1000× *g* for 5 min, discarding the supernatant, twice, to remove the remains of the incubation medium. Finally, the DA in the slices was extracted by adding 0.3 mL of 50% MeOH and frozen at −80 °C. After 1–3 days, the samples were thawed and homogenized in an ultrasonic bath filled with a mixture of water and ice. Finally, the homogenates were clarified by centrifugation at 19,000× *g*, filtered through 0.22 µm syringe filters, and frozen at −80 °C until analysis.

### 4.2. Domoic Acid Uptake Velocity and Saturation of the Transport

This experiment was aimed at checking if the absorption of DA by the digestive gland is linearly dependent (typical of free passage through the cell membrane) or not (typical of the involvement of transporters or carriers) on the concentration of domoic acid in the environment.

The base of the incubation media in this experiment was filter-sterilized (0.22 µm) seawater (salinity = 34). The base medium was supplemented with domoic acid (ABCAM, Cambridge, UK) to obtain concentrations of 5.8, 23.1, 92.3, 369.1 and 1476 µg mL^−1^. Eight slices were incubated in each concentration, and DA extracted following the procedure described above, in this section.

### 4.3. Effect of Environmental Sodium

This experiment was designed to check if the uptake of DA by the digestive gland of the mussel is Na-dependent, as is the case for many amino acid transporters.

Instead of the seawater used in Section 4.2, three artificial seawaters were used. The first one, used as “control”, was prepared by adding 24.55 g of NaCl, 0.75 g of KCl, 4.07 g of MgSO_4_·7H_2_O, 1.47 g of CaCl_2_·2H_2_O, 6.04 g of MgCl_2_·6H_2_O, and 0.21 g of NaHCO_3_ to 1 L of Milli-Q water. The two others (labeled as “choline” and “sorbitol”) did not contain Na. In both of them, NaHCO_3_ was replaced by KHCO_3_, and NaCl was replaced by Choline-Cl (choline medium) and sorbitol (“sorbitol” medium). Domoic acid was added to all incubation media to a 500-µg mL^−1^ level.

Eight slices were incubated in each medium and extracted following the procedure described above, in this section.

### 4.4. Effects of Cyanide in a Chloride-Depleted Environment and pH

This experiment was carried out to check if the uptake of domoic acid was proton-dependent, and as a preliminary evaluation of the effect of cyanide. To evaluate the effect of pH, two aliquots of the incubation media “control” and “choline” of Section 4.3. were used. The pH of one aliquot of each medium was lowered by addition of approximately 25 µL of 85% H_3_PO_4_. Eight slices were incubated in each of the two incubation media (“control” and “choline”), four at each pH treatment (6.5 and 5.1 for the “control”, 7.2 and 5.4 for the “choline” medium). DA was extracted following the procedure described in Section 4.3. To preliminarily evaluate the effect of the metabolic inhibitor cyanide, two aliquots of the remaining treatment (sorbitol) of Section 4.3 were used. NaCN was added to one of them, to a final concentration of 5 mM. Four slices were incubated in each medium, with and without NaCN.

### 4.5. Effect of Environmental Chloride and Cyanide

The aim of this experiment was two-fold. First, to confirm that the transporter was chloride-dependent (discarding a possible effect of sorbitol and the combined effect of Na^+^ and Cl^−^ depletion), and second, to evaluate the effect of cyanide, a metabolic inhibitor, on the uptake in Na^+^- and Cl^−^-sufficient media.

The control treatment was the same as in Section 4.3. For the Cl^−^-free treatment, NaCl was replaced by sodium gluconate (instead of sorbitol) and all other chlorides were replaced by their correspondent gluconates. For the cyanide treatment, a medium made of the same components as the control but with NaCN added to a 5 mM final concentration was used.

### 4.6. LC-MS/MS Analysis

The analysis of DA in the extracts obtained was carried out by LC-MS/MS using a Thermo Accela coupled chromatographic system, through a HESI-II electrospray interface, to a Thermo Quantum Access Max triple quadrupole mass spectrometer (Thermo Fisher Scientific, Waltham, MA, USA).

The chromatographic method used a Kinetex C18 (50 × 2.5 mm, 2.6 µm) reversed-phase chromatographic column (Phenomenex, Torrance, CA, USA), 0.2% formic acid as phase A, and 50% MeOH as phase B. The run started at 100% A, changing linearly to 45% A from min 2 to min 4, maintaining this proportion for 2 min, and then returning in 1.4 min to the initial conditions, which were maintained for 1.6 min to re-equilibrate the column before the next injection. The flow rate was 280 µL min^−1^ and the injection volume 5 µL.

The mass spectrometer was operated in positive ionization mode, with 3500 V of capillary voltage, 20 and 10 nominal units of sheath and auxiliary gas (nitrogen), respectively, 100 °C of capillary temperature, 250 °C of transfer tube temperature, and 1.5 mTorr of collision gas (argon) pressure. The transition 312.1 > 266.1 (collision energy = 15 V) was used for quantification and 312.1 > 248.1 (collision energy = 17) for confirmation.

The quantification was made by the external standard method using solution provided by CIFGA (Lugo, Spain) as a certified reference.

The LOQ (s/n = 10) and LOD (s/n = 3) of the method are 26.3 and 7.9 ng mL^−1^, respectively. They were computed by extrapolation of the s/n obtained for a 39.5 ng mL^−1^ solution, for the peak of the confirmation transition.

### 4.7. Statistical Analysis and Simulation

All statistical analysis and plotting has been carried out with R [57], using the base module and different packages. GGPLOT2 [58] was used for most plots, DRC package [59] for fitting the Michaelis–Menten model, MULTCOMP [60] for post hoc tests, R stats for ANOVA, Student t and Wilcoxon tests, and deSolve [61] for the accumulation simulations (Text S1).

## Data Availability

Not applicable.

## Supplementary Materials

The following are available online at https://www.mdpi.com/article/10.3390/toxins13070458/s1, Text S1—R code for the simulation of domoic acid accumulation. Figure S1—Simulated domoic acid accumulation in King Scallops, as a function of the *Pseudo-nitzschia* cell toxin content.
