# Supplementary material for: Characterization of the Domoic Acid Uptake Mechanism of the Mussel (Mytilus galloprovincialis) Digestive Gland
Source: Toxins (Basel). 2021 Jun 30;13(7):458. doi: 10.3390/toxins13070458 (PMC8310042; doi:10.3390/toxins13070458)
Supplement: Supplementary file 1 [file toxins-13-00458-s001.zip › toxins-1264519-SI.pdf]

Supplementary Materials: Characterization of mechanism domoic acid uptake of the mussel (*Mytilus galloprovincialis*) digestive gland

Juan Blanco, Carmen Mariño, Helena Martín, Araceli E. Rossignoli

Text S1. R Code for the simulation of DA accumulation.

```
library(tidyverse)
library(deSolve)

##Define the differential equation of accumulation
modAcum <- function(tiempo,tox,params){
  with(as.list(c(tox, params)),{
    toxDigest <- ((DAcel_pg/1e6) / (VolPseudo_um3/1e12))*ocupDigest #compute the DA
concentration in the digestive system
    dTox=((Vmax_dia*(toxDigest))/(Km+toxDigest))-tasaDep_dia*Tox #balance of uptake (Michaelis
menten) and depuration
    return(list(c(dTox)))
  })
}

#General information and parameters
simulTodos <- data.frame(matrix(nrow=15,ncol=11)) #define a dataframe to store the results
relacGD_Cuerpo <- 10 #ratio digestive gland weight / body weight
VolPseudo_um3 <-750 # Pseudo-nitzchia cell volume µm3
ocupDigest <- 1 #porportion of the digestive occupied by Pseudo-nitzschia
Km <- 1770 #Estimated Km (Michaelis- Menten)
Vmax_dia <- 79.1*24 #Estimated Vmax (Michaelis- Menten) per day
tiempo <- seq(from=0, to=14) #days of accumulation
tasaDep_dia <- 0.68 # depuration rate day-1

#loop changing DA concentrations per cell
for (i in seq(1,11)) {
  #define the parameters
  DAcel_pg <- 2^((i-5)) #set the DA concentrations per cell to a series power of 2
  parametros <- c(VolPseudo_um3=VolPseudo_um3, #set the general parameters
              DAcel_pg=DAcel_pg,
              ocupDigest=ocupDigest,
              Km=Km,
              Vmax_dia=Vmax_dia,
              tasaDep_dia=tasaDep_dia)
  tox <- c(Tox=0) #Initial DA concentration in the digestive gland
  simul <- as.data.frame(ode(y = tox, times = tiempo, func = modAcum, parms =
parametros))#solve the differential equation
  simulTodos[,i] <-simul$Tox #store the results of the loop in a data frame
}
```

```
#plot the results
simulTodos <- tiempo %>% bind_cols(simulTodos) #add days to the results of the simulation
simulVert <- simulTodos %>% pivot_longer(cols=c(2:12),names_to =
"codToxCel",values_to="concent") #change to long format
names(simulVert)[1] <- "Days"
simulVert$codToxCel <- as.numeric(gsub("X","",simulVert$codToxCel))
simulVert <- simulVert %>% mutate(DAConc_pg_cell=2^((as.numeric(codToxCel)-5))) #add a
column with the DA concentrations per cell
resum <- simulVert %>% group_by(as.factor(DAConc_pg_cell)) %>%  #find the maximum DA
concentration in the digestive gland attained with each DA concentration per cell
  summarise(maximo=max(concent))
maximResum <- resum %>% summarise(maximo=max(maximo))
names(resum)[1] <- "pg_cel"
gr <- simulVert %>%
ggplot(aes(x=Days,y=concent/relacGD_Cuerpo,col=as.factor(DAConc_pg_cell))) #plot DA
concentrations in the whole body
gr+geom_line()+
  geom_hline(aes(yintercept=20), lty=2)+
  geom_text(data=resum,aes(x=14.5,y=maximo/relacGD_Cuerpo,label=pg_cel),col="black")+
  geom_text(data=maximResum,aes(x=14.5,y=(maximo/relacGD_Cuerpo)+20,label="pg~cell^{-
1}"),parse=TRUE,color="black")+
  theme(legend.position = "none",axis.text = element_text(size=12),axis.title =
element_text(size=14))+
  scale_x_continuous(breaks = seq(0,14))+
  scale_y_continuous(breaks = round(seq(min(simulVert$concent)/relacGD_Cuerpo,
max(simulVert$concent)*1.1/relacGD_Cuerpo, by = 50),1))+
  ylab(expression(paste("DA concentration (mg kg "^{-1}~")")))
```

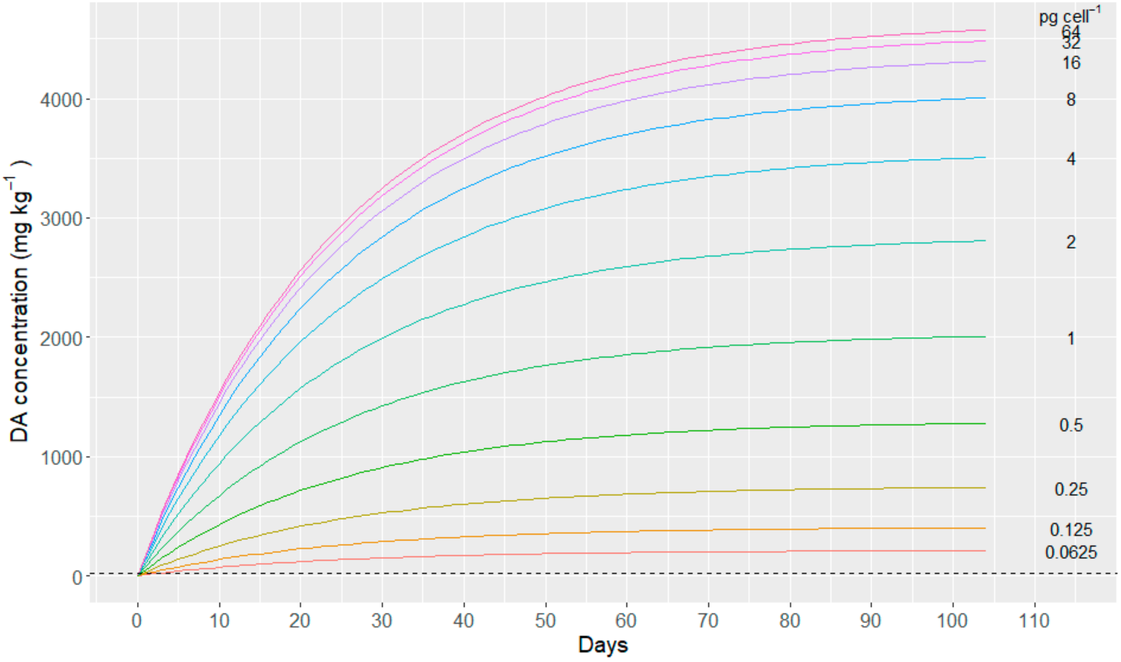

Figure S1. Simulated domoic acid accumulation in King Scallops, as a function of the *Pseudo-nitzschia* cell toxin content, assuming a precise cell volume, that all cells are completely filling the digestive system, and a depuration rate of 0.04 day^{-1}, and the same uptake rates than the mussel *M. galloprovincialis*. The dashed line represents the regulatory level.
